# A MULTI-SITE PROGRAM TO PROVIDE GERIATRICS TELECONSULTATION TO OLDER RURAL VETERANS (GRECC CONNECT)

**DOI:** 10.1093/geroni/igz038.2264

**Published:** 2019-11-08

**Authors:** William Hung, Steven Barczi, cathleen Colon-Emeric, Michelle Rossi, Stuti Dang, Thomas Caprio, Sara Espinoza

**Affiliations:** 1 James J Peters VA Medical Center, Bronx, New York, United States; 2 Geriatric Research, education and Clinical Center, William S Middleton Memorial VA Medical Center, University of Wisconsin; Madison, Madison, Wisconsin, United States; 3 Duke University Geriatric Research; education and clinical center, durham vAMC, durham, North Carolina, United States; 4 Geriatric Research, education and clinical center, VA Pittsburgh healthcare system, pittsburgh, Pennsylvania, United States; 5 GRECC, Miami VAMC, Miami, Florida, United States; 6 Canandaigua VAMC, Canandaigua, New York, United States; 7 GRECC, San antonio VAMC, San antonio, Texas, United States

## Abstract

Older Veterans living in rural areas often do not have access to geriatrics team care; rural frontline providers and teams may need support to address the needs of older adults with complex chronic conditions. GRECC Connect aims to link up geriatric teams at Geriatric Research, Education and Clinical Centers (GRECCs) and rural clinics to provide geriatric consultation remotely through clinical video telehealth (CVT) and other means. GRECC Connect is established in twelve GRECCs across the country with links to rural clinics in their catchment area; consultations led to identification and meeting of care needs of older adults with complex conditions, improving medication use and reducing older adults’ need for travel to long distances for consultation. In this presentation, we review the experience of establishing connections with rural clinics, impact on older adult care and adaptations needed to address local needs and contexts.

